# Mask ventilation

**DOI:** 10.12688/f1000research.15742.1

**Published:** 2018-10-23

**Authors:** Paul Baker

**Affiliations:** 1 Department of Anaesthesiology, University of Auckland, Auckland, New Zealand; 2 Starship Children’s Hospital, Auckland, New Zealand

**Keywords:** Mask ventilation

## Abstract

Effective mask ventilation is an essential skill for any practitioner engaged in airway management. Recent methods to objectively describe mask ventilation using waveform capnography help practitioners to monitor and communicate the effectiveness of mask ventilation.

Gentle mask ventilation is now considered acceptable during rapid sequence induction/intubation after loss of consciousness, hence reducing the incidence of hypoxia prior to tracheal intubation. Mask ventilation can be enhanced with muscle relaxation, a double C-E grip, and jaw thrust. This is particularly relevant for patients with reduced apnoea time.

An awareness of the complications associated with mask ventilation may help reduce the morbidity associated with this technique. Effective ventilation technique and optimum device selection are important aspects for resuscitation of the newborn. Teaching correct establishment and maintenance of mask ventilation is essential for safe patient care.

This review will examine some of the latest developments concerning mask ventilation for adult and paediatric patients.

## Describing mask ventilation

Given the significance of mask ventilation (MV), it is important to be able to measure and objectively describe the outcome of this procedure. Previous attempts to do this have been largely subjective, complicated, and rarely applied. In a Japanese Society of Anaesthesiologists airway management guideline, it was proposed that three distinct capnogram waveforms could be used to grade ventilation during MV ^1^. The latest method to address this problem has also applied waveform capnography as an objective measure of adequate alveolar ventilation ^2^. A four-point scale has been designed which grades the quality of mask ventilation according to the pattern and magnitude of the capnography.

Grade A includes a large end-tidal carbon dioxide (ETCO _2_) wave and plateau. Grade B has no plateau and a smaller wave with ETCO _2_ at least 10 mmHg. Grade C has no plateau and ETCO _2_ <10 mmHg, and grade D has no ETCO _2_. Objective capnograph findings are supplemented with a check box which includes Guedel airway, one- or two-handed technique, and nasopharyngeal airway. These details help to explain how the difficult MV was managed. Waveform capnography can therefore be used as a monitor of effective mask ventilation and also a method to communicate relative difficulty in a similar way to the Cormack-Lehane scale used to record laryngoscopy. Another helpful objective measure of MV is spirometry. Both inspiration and expiration can be measured with flow volume loops. This facility is available on many anaesthetic machines.

## Rapid sequence induction/intubation

In 1970, a fifteen-step method of rapid sequence induction and intubation (RSII) was described to avoid aspiration of gastric contents and other debris during emergency airway management ^3^. Since that time, this technique has been modified and criticised ^4^. In the original RSII, MV was recommended for pre-oxygenation, but this was discontinued following induction of anaesthesia until tracheal intubation and inflation of the tube cuff.

It is now known that some patients can become hypoxic during the apnoea phase between pre-oxygenation and tracheal intubation ^5^. The incidence of hypoxia during RSII can be as high as 35.9% (SpO _2_ <95%) ^6^. Hypoxia during this phase is particularly prevalent in those individuals with reduced apnoea tolerance including infants, obese patients, and pregnant women. Oxygen desaturation in overweight patients during RSII was also seen earlier in patients receiving succinylcholine versus rocuronium. This observation was explained by increased metabolism secondary to succinylcholine fasciculations ^5^.

The authors’ opinion from the 2015 Difficult Airway Society practice guideline for the management of unexpected difficult tracheal intubation in adults was that gentle MV prior to tracheal intubation is recommended to prolong apnoea time without hypoxia ^7^. This is consistent with a 1987 study of MV during apnoea that demonstrated no gas in the stomach with gentle MV, only half of patients suffering gastric gas with maximal inflation, and no gas in the stomach with cricoid pressure on 8. The issue of cricoid pressure is still unresolved, with growing scepticism about the effectiveness of cricoid pressure to reduce gastric distension and avoid aspiration. Cricoid pressure can make MV difficult. If this occurs, the cricoid pressure should be released.

## Mask ventilation during anaesthetic induction

Immediately after the intravenous injection of anaesthetic drugs, MV becomes necessary to support ventilation and avoid potential airway obstruction. It is common practice for an anaesthetist to apply a face mask to the patient’s face with one hand while squeezing an anaesthetic circuit ventilation bag with the other hand. Similarly, a self-inflating bag is often operated with only one hand applied to the face mask.

In a study comparing MV in normal patients and patients with multiple risk factors for sleep disordered breathing (SDB), Sato *et al*. found that expiratory flow limitation (EFL) and obesity independently explained reduced MV efficiency ^9^. It was also found that resorting to two-handed MV effectively restored inefficient MV. Sato also found that tidal volume during one-handed MV increased by more than 70% during the first minute. This has been attributed to deepening of anaesthesia and the onset of neuromuscular blockade, but other physiological mechanisms could also help to explain this observation. Interaction between lung volume and pharyngeal collapse and increased chest wall rigidity secondary to opiate administration could contribute to difficult MV during anaesthesia, particularly in patients with SDB and obesity. Clinically, obesity, lingual tonsils, and patients with a history of SDB are important to detect preoperatively in an effort to decrease difficult MV ^9– 11^. Obesity was identified as one of the risk factors for difficult and impossible MV ^12, 13^. Correct positioning may improve conditions for effective mask ventilation by adopting anti-Trendelenburg or Fowler positions to reduce abdominal pressure on the diaphragm.

MV generally improves after the onset of muscle relaxation. Patients with distal airway obstruction, including tracheomalacia and mediastinal masses, are rare exceptions and should not be paralysed. In these cases, paralysis can lead to extrinsic large airway compression, elimination of diaphragmatic movement, and decreased expiratory flow. The practice of testing the quality of MV prior to the administration of neuromuscular blockers is no longer supported ^14^.

Maintaining a patent airway during an inhalation induction of anaesthesia can be particularly challenging in infants with micrognathia. Optimum MV techniques may be required in these patients. This technique includes two people: one ventilates the bag using 100% oxygen and a respiratory rate consistent with the resting respiratory rate of the child. A double C-E grip with fingers on the jaw and mask helps to create a jaw thrust and minimises leaks around the mask. Ventilation can then proceed with an assistant squeezing the bag or the use of a pressure-controlled ventilator ^15^. Airway manoeuvres include jaw thrust, head tilt, and chin lift. Of these, jaw thrust is the most effective to open the obstructed airway in an anaesthetised child ^16^.

## Complications arising from mask ventilation

Mask ventilation is regarded as the cornerstone of airway management and the position of safety when other airway techniques fail. In the Difficult Airway Society 2015 guidelines for the management of unanticipated difficult intubation in adults, step 3 applies to MV, when tracheal intubation and supraglottic airway insertion have been attempted and failed ^7^. Yet, despite the importance of MV, there are reports of complications arising from MV and known incidences of difficult and impossible MV for adults and children during anaesthesia.

In a prospective study of 22,660 MV attempts, Kheterpal *et al*. found a difficult MV incidence of 1.4% (1:71) ^12^. Difficulty was defined as grade 3 on a four-point scale of MV difficulty defined by Han *et al*. ^17^. A subsequent study by Kheterpal *et al*. of 53,041 patients found an incidence of 0.15% (1:666) impossible MV ^13^. Unexpected difficult MV in a group of 484 children was 6.6% ^18^.

Various complications have been reported with MV. Difficulty achieving a seal with the face mask can be attributed to edentulous facies. Replacing the dentures or packing the sides of the face around the facemask are simple solutions ^19^. The presence of a beard may create large gas leaks around the face mask. This problem can be pre-empted by shaving the beard or applying an occlusive dressing with a hole cut in it for the mouth. A case of difficult facial morphology, secondary to a frontonasal encephalocele in a term infant, created difficult conditions for MV. This was overcome with a novel solution by using an inverted adult size five face mask over the infant’s face ^20^.

Aspiration is a common complication of MV which can be obviated, if possible, by observing fasting guidelines or using a gastric tube to drain the stomach before airway management. Application of cricoid pressure may also help reduce gastric distension during MV, but caution is required because cricoid pressure may also make MV difficult.

Inability to oxygenate and ventilate during MV can improve with head up positioning, administering a neuromuscular blocker, and the use of oropharyngeal and nasopharyngeal airways, jaw thrust, and two-handed mask ventilation ^14, 16, 21^.

Poor MV technique can have adverse physiological consequences, particularly during emergency airway management. For example, hyperventilation decreases PaCO _2_, leading to vasoconstriction. Small tidal volumes (6–7 ml/kg) and a short inflation duration of 1 second are recommended and help to avoid rapid or forceful breaths ^22^.

Mechanical failure of a non-rebreathing self-inflating bag can cause patient morbidity. There are multiple components to a self-inflating bag including a cuffed mask, non-rebreathing patient valve, self-inflating ventilation bag, air-intake valve, oxygen nipple for oxygen tubing, oxygen reservoir bag, and a reservoir system pressure release valve ^23^. Each of these components can potentially fail. For example, a case of a faulty non-rebreathing patient valve led to excessive positive end expiratory pressure (PEEP) and inspiration without expiration, causing excessive intrathoracic pressure. The implications of this included decreased lung compliance, high inspiratory pressure, high airway resistance, and a pneumomediastinum. As a consequence of these changes, decreased venous return can lead to decreased cardiac output and a false-negative capnograph can also occur, resulting in a false diagnosis of oesophageal intubation ^23^.

## Neonatal mask ventilation

Up to 10% of newborn infants receive some form of respiratory support at birth. Current American and European resuscitation guidelines recommend the administration of positive pressure ventilation (PPV) to treat apnoea in preterm and term infants ^24, 25^. Options to administer PPV include a flow-inflating or self-inflating resuscitation bag or a T-piece resuscitator device. There is little comparative evidence available to evaluate the merits of these devices. Two randomised controlled trials in newborn infants compared the T-piece resuscitator and a self-inflating bag. One examined short- and intermediate-term morbidity and mortality outcomes and found no difference between the two devices ^26^. The other study found that both a mask leak and airway obstruction were common during MV with PPV using a T-piece and a self-inflating bag. These problems can be recognised and corrected by using a respiratory function monitor (RFM) which displays gas flow, tidal volume, and pressure waves ^27^.

A study using lung models compared the Ambu self-inflating bag and the Neopuff T-piece resuscitator. Five experienced clinicians ventilated preterm, term, and infant lung models. Compliance, delivery pressure, and inflation rates were measured. The Ambu SIB accurately delivered targeted pressures to all three models, but it was not possible to deliver targeted pressures to the infant model with the Neopuff infant resuscitator ^28^.

A clinical study of MV in preterm infants used an RFM to compare spontaneous breaths and PPV using a round silicone face mask and a T-piece device. Tidal volume, peak inspiratory pressure, peak end expiratory pressure, and mask leak were measured. It was found that large leaks occurred around the face mask and high tidal volumes occurred during PPV in contrast to smaller volumes found during spontaneous breathing with CPAP ^29^.

A recent prospective cohort study involving 1,962 premature neonates compared PPV at birth with a T-piece resuscitator or self-inflating MV without PEEP. Outcome measures included survival to hospital discharge without bronchopulmonary dysplasia, severe peri-intraventricular haemorrhage, and periventricular leukomalacia. The study showed that the T-piece resuscitator resulted in patients being more likely to survive to hospital discharge while avoiding major morbidities ^30^.

## Training mask ventilation

MV is an important clinical skill which requires training and practice with good technique. An optimum MV technique has been devised based on a literature review and the development of a conceptual framework. This approach addresses MV in terms of inadequate mask seal, increased airway resistance, and decreased respiratory compliance. Techniques can be taught to address difficulties in each of these categories ^31^.

Learning curves are more rapid and less variable for MV than for tracheal intubation. In one study, novice interns achieved a 20% failure rate or better after a median of 25 procedures ^32^. Trainee participants in a paediatric context demonstrated improved tidal volumes and inspiratory pressures when practicing two-person compared to one-person MV ^33^. Training accurate respiratory rates during paediatric MV can be improved with the use of a metronome ^34^.

## Conclusion

This review of MV emphasises current approaches to this important clinical skill. MV is a core skill in airway management and is the default technique when all others fail. Successful MV requires good technique and regular practice.
